# piR-19166 inhibits migration and metastasis through CTTN/MMPs pathway in prostate carcinoma

**DOI:** 10.18632/aging.103677

**Published:** 2020-09-03

**Authors:** Tingyue Qi, Haiyan Cao, Hongguang Sun, Hao Feng, Nianfeng Li, Chenghai Wang, Lei Wang

**Affiliations:** 1 Department of Ultrasound, Medical Imaging Center, Affiliated Hospital of Yangzhou University, Yangzhou University, Yangzhou 225009, China; 2 Department of Pathology, Affiliated Hospital of Yangzhou University, Yangzhou University, Yangzhou 225009, China

**Keywords:** piRNAs, piR-19166, prostate carcinoma, CTTN, metastasis

## Abstract

Tumor metastasis is one of death causes for patients of prostate carcinoma. PIWI-interacting RNAs (piRNAs) are a subtype of noncoding protein RNAs that are involved in tumorigenesis, but the effect of piRNAs in prostate carcinoma (PCa) remains unclear. This article showed the identification of piRNAs was performed using a piRNA microarray screen in PCa tissues and several piRNAs were identified as dysregulated. The two up-regulated piRNAs (piR-19004 and piR-2878) and one down-regulated piR-19166 have been validated in the tissues and cell lines of PCa using quantitative reverse transcription polymerase chain reaction (qRT-PCR). Further studies showed that piR-19166 is transfected into PCa cells to suppress its migration and metastasis. Mechanistically, cortactin (CTTN) 3' untranslated region (UTR) was complementary combined with piR-19166 by bioinformatic prediction and identified as a direct target of piR-19166 through dual-luciferase reporter assay. Over-expression and knockdown of CTTN could respectively rescue and simulate the effects induced by piR-19166. Finally, piR-19166 suppresses migration and metastasis by the CTTN/matrix metalloproteinases (MMPs) pathway in PCa cells. Thus, these findings suggested that piR-19166 targets the CTTN of prostate cancer cells to inhibit migration and distant metastasis, and may represent a new marker of diagnosis and treatment for PCa patients in early stages.

## INTRODUCTION

Prostate carcinoma (PCa) is a common form of malignant tumor in older men and is the second leading cause of cancer-associated death in United States [1, 2]. Although the combination of earlier stage of PSA testing and advances in treatments [3, 4] has effectively prolonged the survival time of PCa patient, it is difficult to completely prevent distant metastasis and recurrence of PCa. Though lymphatic metastasis and hematogenous metastasis is two of the common metastasis pathways for prostate carcinomas, the mechanism of metastasis of prostate cancer is still unclear.

Recently, the functions of noncoding protein RNAs (ncRNAs) in PCa have been widely studied [5–9]. PiRNAs are a subtype of small noncoding protein RNAs (sncRNAs) and aberrant expression of piRNAs has been reported as crucial regulators for tumorigenesis of tumor [10, 11]. But the effect of piR-19166 in prostate carcinoma remains unclear. Therefore, it is necessary to explore the regulatory mechanism of piR-19166, which may provide insight into novel marker of diagnosis and treatment for PCa patients in early stages.

In the current study, piR-19166 was down-regulated by qRT-PCR in PCa tissues and cell lines, and correlated negatively with metastasis. In addition, the function of piR-19166 in migration of PCa cells, the target of piR-19166 and metastasis mechanism of PCa were investigated together. This study may presents piR-19166 served as a novel target for further early therapeutic studies of PCa.

## RESULTS

### PiRNA screen and data analysis

**Figure 1:**
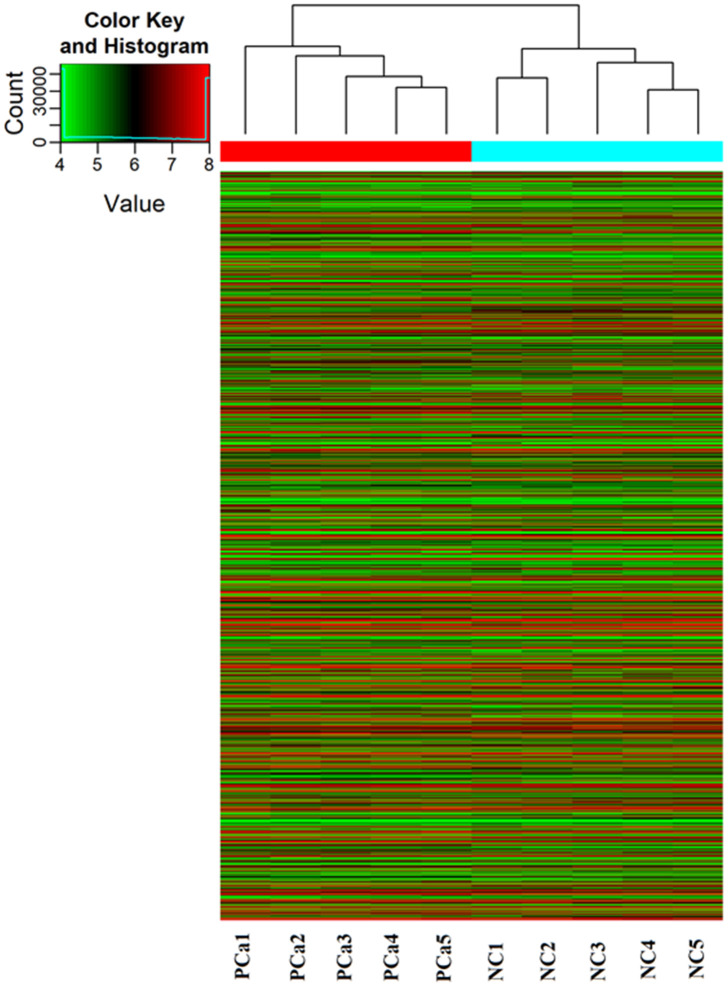
**PiRNA expression profiling in Arraystar piRNA Microarray.** The Hierarchical Clustering shows a distinguishable piRNA expression profiling among samples. “Red line” indicates high expression, and “Green line” indicates low expression. Five PCa tissues (experimental) and five corresponding normal tissue (NC) were used to perform the piRNA microarray in triplicate.

**Figure 2:**
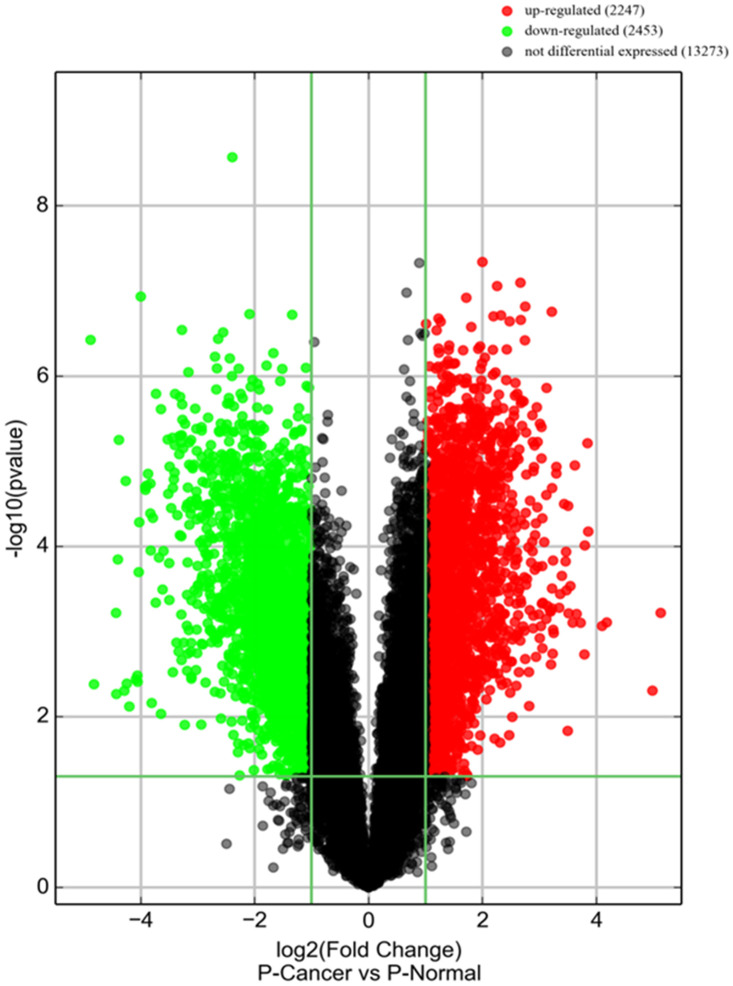
**piRNA expression in PCa tissues.** Volcano plot shows piRNA differential expression in PCa tissues and controls using fold-change values and P-values. The horizontal green line represents a P-value of 0.05, and the vertical green lines correspond to 2.0-fold up and down, respectively. “Red points” indicates high relative expression and “Green points” indicates relative low expression with statistical significance (P<0.05).

A piRNA expression profiling of Arraystar piRNA Microarray was conducted using RNA isolated from five PCa tissues and five normal prostate tissues (NC). The screen and data analysis were performed by Arraystar Inc. The results showed that a box plot (Figure 1) formed a normalized log2-ratio distribution of intensities between the PCa group and NC group; a Volcano Plot filtered between the experimental and control groups with a threshold fold change > = 2.0 and p-value < = 0.05 was performed (Figure 2) to identify differentially expressed piRNAs with statistical significance. Hundreds piRNAs were aberrant expression that appear to up-regulated and down-regulated in this data.

### piRNA validation in PCa tissues

**Figure 3:**
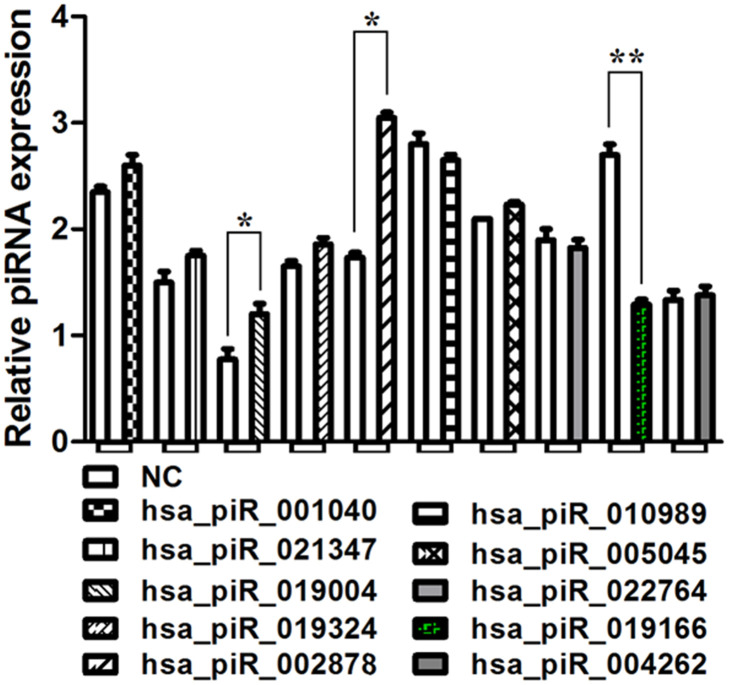
**qRT-PCR analysis of select piRNAs.** Validation of selected piRNA by qRT-PCR in 42 prostate cancer tissues compared to march normal prostrate tissues. The expression of hsa_piR_019004 (piR-19004) and hsa_piR_002878 (piR-2878) were up-regulated while hsa_piR_019166 (piR-19166 or piR19166) was down- regulated in PCa tissues. *P < 0.05, **P < 0.01.

Several piRNAs identified by the piRNA screen were up-regulated or down-regulated between PCa group and NC group, their expression folds were listed in Table 1. The expression of three of these piRNAs has been validated confirmedly by qRT-PCR in 42 pairs of PCa specimens and matched normal prostate tissues. The expression of hsa_piR_019004 (piR-19004) and hsa_piR_002878 (piR-2878) were up-regulated while hsa_piR_019166 (piR-19166) was down- regulated in PCa tissues (Figure 3). According to this result, down-regulated piR-19166 was only selected to do the research object of future experiment.

**Table 1:** Selected piRNA identified using piRNA array screen in PCa.

**Up-regulated piRNAS**		**Down-regulated piRNAs**	
**PiRNA**	**Fold Change**	**PiRNA**	**Fold Change**
hsa_piR_001040	17.1	hsa_piR_010989	-28.3
hsa_piR_021347	13.2	hsa_piR_005045	-21.5
hsa_piR_019004	12.6	hsa_piR_022764	-16.6
hsa_piR_019324	9.3	piR-19166	-12.1
hsa_piR_002878	8.3	hsa_piR_004262	-9.0
hsa_piR_019014	7.7	hsa_piR_016735	-6.0

### Expression of piR-19166 is down-regulated in PCa tissues and cell lines

**Figure 4:**
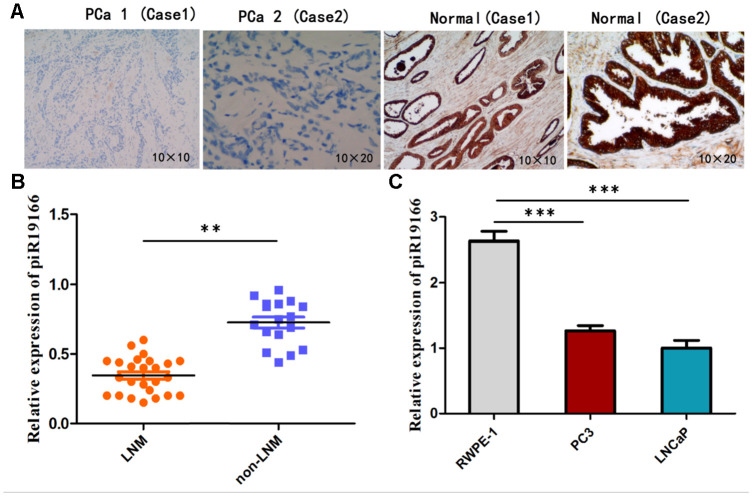
**Expression of piR-19166 in PCa tissues and cell lines.** (**A**) ISH showed expression of piR-19166 was negative (30/42) compared with the adjacent normal tissues (positive, 35/42). (**B**) Compared with the negative group of lymph node metastasis (LNM), a significantly low level of piR-19166 was detected in the LNM -positive group by qRT-PCR (Pearson Chi-Square=42.000, **P<0.01). (**C**) Down-regulation of piR-19166 was detected using qRT-PCR in PC3 (T test, t=33.15, ***P < 0.001) and LNCaP (T test, t=32.05, ***P < 0.001) compared with normal prostate epithelial cell RWPE-1. *P < 0.05, **P < 0.01, ***P < 0.001.

To further confirm down-regulated expression of piR-19166, RNA in situ hybridization (ISH) was explored its RNA expression in 42 specimens of PCa and matched normal prostate tissues, and qRT-PCR was detected in PCa cell lines(PC3 and LNCaP) and RWPE-1(Human Prostate Epithelial Cell). As shown in Figure 4A, expression of piR-19166 was negative (30/42) compared with the adjacent normal tissues (positive, 35/42) via ISH in most specimens. By qRT-PCR, expression of piR-19166 in PCa tissues was obviously lower than that of in the adjacent normal tissues (Figure 3). Furthermore, compared with the negative group of lymph node metastasis (LNM), a significantly low level of piR-19166 was detected in the LNM -positive group using qRT-PCR (Figure 4B). There is closely relation between expression of piR-19166 and LNM (Pearson Chi-Square=42.000, p < 0.01). Similarly, down-regulation of piR-19166 was detected using qRT-PCR in PC3 (T test, t=33.15, P < 0.001) and LNCaP (T test, t=32.05, P < 0.001) cell lines compared with normal prostate epithelial cell RWPE-1 (Figure 4C). Collectively, the above findings suggest that piR-19166 expression may be an inhibitor in development and metastasis of PCa.

### piR-19166 inhibited migration and metastasis in PCa cell lines

**Figure 5:**
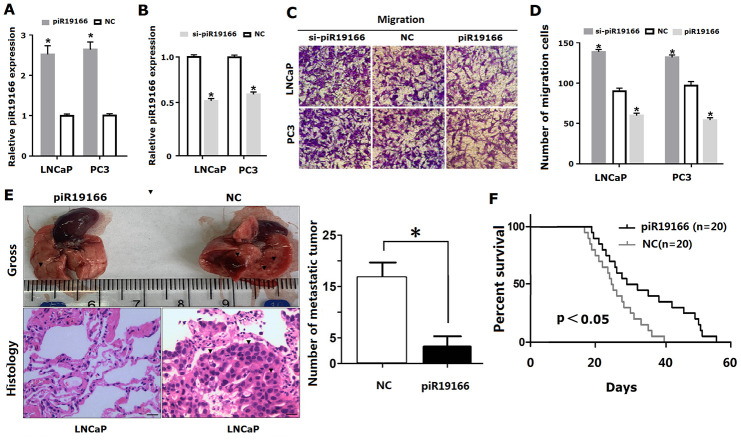
**PiR-19166 suppressed migration and metastasis in PCa.** (**A**, **B**) Overexpressing or silencing of piR-19166 was confirmed into PC3 and LNCaP cells by qRT- PCR (*P<0.05). (**C**, **D**) The cell migration of PC3 and LNCaP was assessed by Transwell assay (*P<0.05). (**E**) The number of metastatic tumor (blue arrow) was assessed by assay of lung metastasis models of nude mice after overexpression of piR-19166 in LNCaP cells (*P<0.05). (**F**) The survival time was assessed between overexpression group of piR-19166 and control group (NC),*P<0.05.

The above data demonstrated piR-19166 was relation closely with LN metastasis in PCa patients, so the role of piR-19166 will be investigated in migration though Transwell Assay. Firstly, lentiviral vector overexpressing or silencing of piR-19166 and corresponding negative control (NC) was transfected and confirmed into PC3 and LNCaP by qRT- PCR analysis (P<0.05, Figure 5A, 5B). Then, the cancer cells were used for migration. The results of migration showed overexpression of piR-19166 significantly impeded migration in PCa cells, but silencing of piR-19166 dramatically promoted the migration of PC3 and LNCaP (Figure 5C, 5D). Both groups had significant statistical significance (P<0.05). To further investigate the role of piR-19166 in driving PCa metastasis, lung metastasis models of nude mice were also conducted. LNCaP cells with stably overexpressed piR-19166 were injected through the tail vein as compared with controls. The test result showed a significant difference between the piR-19166-overexpressed animals and control groups in macroscopic observation of lung metastasis (Figure 5E). Furthermore, Histologic analysis revealed significantly more and larger metastatic foci in the harvested lung tissues of nude mice injected with PC3 cells with overexpressed piR-19166 (Figure 5E, P<0.05). In addition, in mice xenograft, the survival time of overexpression group of piR-19166 was longer than that of NC (Figure 5F, P<0.05). Collectively, these results suggested that piR-19166 serves as a tumor suppresser in migration of PCa and a role of inhibitor for piR-19166 in the regulation of metastasis in PCa.

### CTTN is a direct target of piR-19166

**Figure 6:**
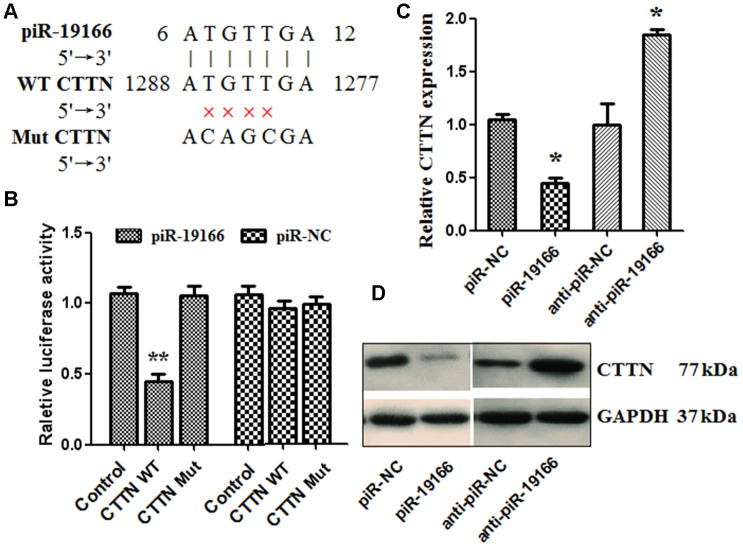
**piR-19166 targeted directly EPHA2 in PC3 cells.** (**A**) DNA binding site sequence between piR-19166 and the 3′-UTR of CTTN and the sequence of wild type (WT) or mutant type (MT) were showed. (**B**) The effect of piR-NC and piR-19166 on the activity of the luciferase reporter containing either WT or MT was detected by dual-luciferase reporter assay. (**C**, **D**) RNA and protein levels of CTTN were, respectively, tested by qRT-PCR and western blot in PC3 cells transfected with piR-19166 or anti-piR-19166 compared with those treated with negative control (NC). *P <0.05, **P <0.01.

CTTN gene was a novel target of piR-19166 by Go Analysis of Arraystar piRNA Microarray and bioinformatics prediction (https://blast.ncbi.nlm.nih.gov/ Blast.cgi?PAGE_TYPE=BlastSearch). The bioinformatics comparison result also showed there is one complementary binding site between piR-19166 and CTTN 3’UTR region (Figure 6A). Duel-luciferase reporter assays were conducted to verify whether piR-19166 directly targeted CTTN. The binding sequences of piR-19166, CTTN of wild type (WT) and CTTN of mutation type (MuT) were showed in Figure 6A. Co-expression with CTTN -3′UTR/pGL3-BS and piR-19166 in PC3 cells caused significant decrease in the luciferase activity compared with the negative control, but this repressive effect disappeared by mutation CTTN (p < 0.01, Figure 6B). This result indicated that piR-19166 exerts inhibitory effects on CTTN expression via binding with the 3′UTR of CTTN. Meanwhile, overexpression of piR-19166 suppressed CTTN expression, and knockdown of piR-19166 promoted CTTN expression in levels of RNA and protein (Figure 6C, 6D). These data indicated that piR-19166 might suppress CTTN expression in a way that prevented post-transcriptional translation of CTTN.

### PiR-19166 exerts its effect on the regulation of CTTN

**Figure 7:**
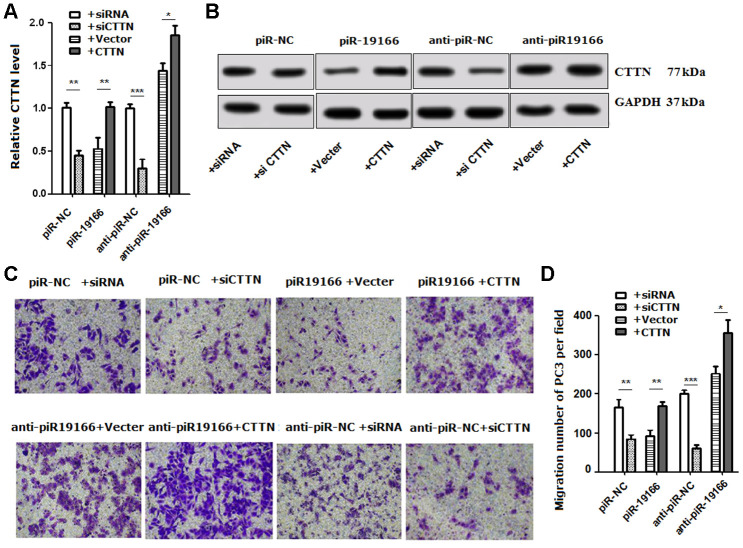
**Rescue assays detected the effects of CTTN suppressed by piR-19166 in metastasis.** (**A**, **B**) RNA and protein levels of CTTN were detected respectively by qRT-PCR and western blot in PC3 cells with the presence of CTTN overexpression or vector control and si-CTTN or siRNA control. (**C**, **D**) Overexpression of CTTN rescued the biologic effects via piR-19166-induced, whereas knockdown of CTTN simulated the effects associated with piR-19166 through cell migration in PC3. Error bars represent the mean ± SD of three independent experiments. *P <0.05, ** P < 0.01, ***P < 0.001.

Rescue assays were conducted to determine whether CTTN was suppressed though piR-19166-induced in PCa cells. Firstly, Over-expression CTTN rescued the down-regulation of CTTN via high levels of piR-19166 (Figure 7A, 7B). Further functional studies confirmed that over-expression of CTTN could provoke piR-19166 and anti-piR-NC mediated suppression of migration, whereas silencing CTTN could abrogate piR-NC and anti- piR-19166 abilities to induce migration (Figure 7C–7E). Collectively, these results indicated that piR-19166 inhibited CTTN expression and then hindered migration in PCa cells.

### PiR-19166 inhibits metastasis though CTTN /MMPs pathway in PCa

**Figure 8:**
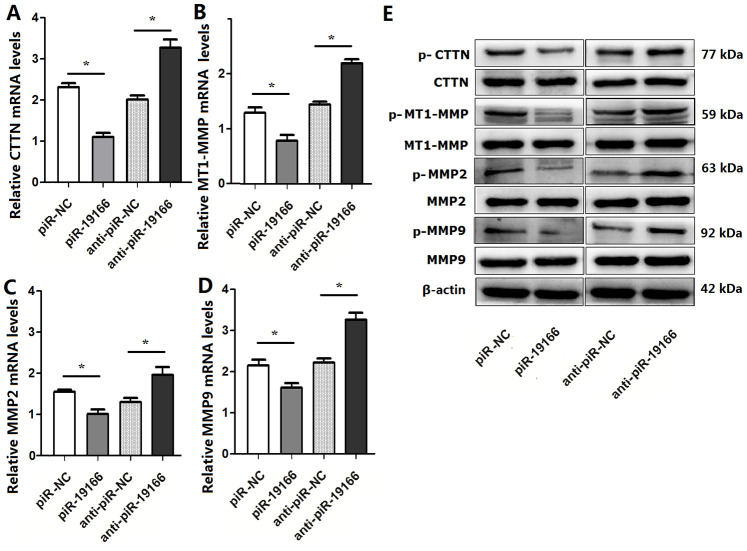
**piR-19166 inactivated CTTN /MMPs pathways in PC3 cells.** (**A**) mRNA levels of CTTN were detected in PC3 of piR-19166 overexpressing or silencing compared with the negative control (NC) by qRT-PCR. (**B**) mRNA levels of MT1-MMP were detected in PC3 of piR-19166 overexpressing or silencing compared with NC by qRT-PCR. (**C**) Levels of MMP2 mRNA were detected in PC3 of piR-19166 overexpressing or silencing compared with NC by qRT-PCR. (**D**) mRNA levels of MMP9 were detected in PC3 of piR-19166 overexpressing or silencing compared with NC by qRT-PCR. (**E**) Protein levels of p-CTTN, p-MT1-MMP, p-MMP2 and p-MMP9 were detected in PC3 of piR-19166-overexpressing or piR-19166-knockdown compared with NC by western blot. Error bars represent the mean ± SD of three independent experiments. *P <0.05.

**Figure 9:**
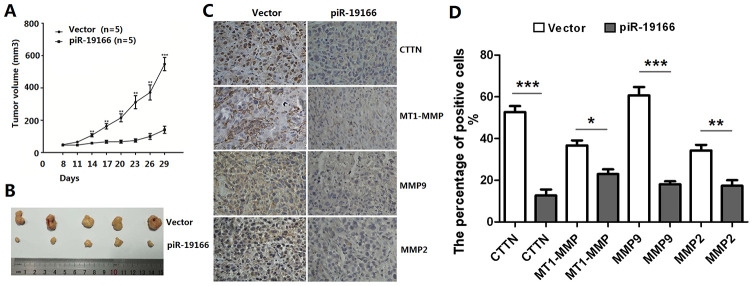
**piR-19166 suppressed CTTN/ MMPs signaling pathways of PC3 *in vivo*.** (**A**) piR-19166 overexpression remarkably inhibited the tumor volumes *in vivo*. (**B**) After 30 days post-injection, all mice were sacrificed, and subcutaneous tumors were collected. (**C**-**D**) The Immunohistochemistry (IHC) results showed that overexpression piR-19166 of xenograft tumor had distinctly lower the percentage of positive cells of p-CTTN, p-MT1-MMP, p-MMP2 and p-MMP9 than those of the control group. Error bars represent the mean ± SD of three independent experiments. *P <0.05, **P <0.01, ***P <0.001.

Previous evidence has suggested that matrix metalloproteinases (MMPs) include MT1-MMP, MMP2 and MMP9, are a direct target of CTTN [14], the CTTN /MMPs pathways are involved in invadopodia and metastasis. So, whether the CTTN /MMPs pathways could be activated by piR-19166, qRT-PCR and western blot assay were used to test this effect after piR-19166 overexpression or knockdown. These results showed that CTTN, MT1-MMP, MMP2 and MMP9 were obviously decreased in high piR-19166 groups rather than those with low piR-19166 groups in mRNA (Figure 8A–8D) and protein (Figure 8E) level. Therefore, this study was concluded that the piR-19166 suppressed expression of CTTN, and then inhibited MMPs signaling pathways might work in PCa cells. Xenograft experiments further revealed that the volume of overexpression of piR-19166 were significantly less than their parent cells (N=20) in nude mice (Figure 9A, 9B, P < 0.01). And at the end of experiment, the xenograft cancer cells generated overexpression of piR-19166 of were stained by immunostaining of anti-CTTN anti-MT1-MMP, anti-MMP2 and anti-MMP9, which showed that the percentage of positive cells were much lower than blank control cells using BN-880a pathological image analysis system (Shanghai Hanfei Medical Instrument Co., Ltd., Shanghai, China) (Figure 9C, P < 0.01). Therefore, these data also demonstrated that the piR-19166 overexpression significantly suppresses CTTN/MT1-MMP/MMP2/MMP9 signaling pathways *in vivo* to regulate metastasis of PCa.

## DISCUSSION

As a primary prostate tumor, its prognosis is relatively favorable and 5-year survival rate in the worldwide is more than 98 % [15]. By reason of the recurrence and metastasis of prostate tumor, there are still no effective drugs for the treatment of prostate tumor. Clinically, pathologic biopsy can not only confirm the diagnosis of prostate cancer, but also evaluate the extent of metastasis after surgery operation. The current treatment for the disease is a combination of surgery, hormone therapy, radiotherapy and chemotherapy [16]. Therefore, insightful understanding into the development mechanism underlying this cancer is still in urgent need for better therapy purpose.

PIWI-associated RNAs (piRNAs) belongs to non-coding protein small RNA with the length of 25 to 33nt, playing a biological role by interacting with PIWI protein. There are four kinds of PIWI protein in human, including PIWIL1 / HIWI, PIWIL2 / HIWI, PIWIL3/ HIWI and PIWIL4 / HIWI. Gene alteration in cancer cells are regulated by a series of small molecules, including piRNAs. Previous research results explored that PIWI protein and piRNAs are related to development and progression of cancer. For example, PIWI protein has abnormal expression in a variety of tumors, such as seminoma, gastric cancer, breast cancer, pancreatic cancer, liver cancer, glioma and so on, which may play a role of carcinogen or tumor suppressor [17–20]. PiRNA also plays the role of oncogene or tumor suppressor gene in tumor formation. PiR-651, piR-4987, piR-20365, piR-20485 and piR-20582 were highly expressed in breast cancer [21]. The expression of piR-823 decreased in gastric cancer, and the level of piR-823 was positively correlated with the stage of gastric cancer [22]. Law et al. [23] reported that the level of pirhep1 was positively correlated with the invasion and metastasis of hepatoma cells, indicating that piRNA might also participate in the invasion and metastasis of tumor cells and other pathological processes. The abnormal high expression of piR-594040 was found in bladder cancer [24]. PIWI and piRNA are closely related to cancer, but there are few studies on the mechanism of piR-19166 regulating development and progression of prostate cancer.

In the current study, piR-19166 was obvious down-regulation in PCa tissues and cell lines compared to adjacent normal prostate tissues and normal prostate epithelial cells via ISH and qRT-PCR, and the piR-19166 expression in lymph node metastasis (LNM) group is lower than that of in non lymph node metastasis group by qRT-PCR. These data demonstrated piR-19166 may be a tumor suppressor gene in development of PCa. Besides, close relation between piR-19166 expression and LNM hinted PCa patients with negative expression of piR-19166 were more prone to lymph node metastasis. Similarly, *in vitro* suggested that piR-19166 could block cell metastasis of PCa cells and decrease the number of metastatic tumors in lung of nude mice. The observations explored piR-19166 was served as a biomarker of metastasis in PCa.

CTTN has been confirmed as an oncogene and positive regulator of metastasis by previous study [25–27]. By bioinformatics predict that CTTN was a target gene of piR-19166 and there is one complementary binding site between piR-19166 and CTTN 3’UTR region. Our arrays showed piR-19166 could inhibit directly protein translation of CTTN via 3’UTR region. These data confirmed that piR-19166 is one of direct inhibitor of CTTN, and piR-19166/CTTN axis involved in metastasis of PCa. Previous considerable research has suggested that a number of CTTN signal pathways are involved in the formation and development of tumors [28–30]. Our results only confirmed CTTN/MMPs signal pathway was valid, that the piR-19166 overexpression significantly suppressed the expressions of CTTN, MT1-MMP, MMP2 and MMP9 at mRNA and protein level in PCa cells, meanwhile, the knockdown of piR-19166 sharply promoted the expressions of CTTN, MT1-MMP, MMP2 and MMP9 in PCa cells. These results found that the piR-19166/CTTN axis activated CTTN/MMPs signaling pathways and prevented migration and metastasis of PCa cells. These are important markers of personalized therapeutics for early-stage PCa patients with LN metastasis.

In summary, low levels of piR-19166 are associated with LN metastasis in PCa; piR-19166 may suppress migration and metastasis by regulating CTTN/ MT1-MMP/MMP2 /MMP9 signaling pathway. The newly identified results suggest that piR-19166 is an important tumor suppressor gene and block- metastatic gene in PCa. The research could provide a new diagnostic and therapeutic target for PCa patients with lymph node metastasis.

## MATERIALS AND METHODS

### Prostate carcinomas and matched normal tissues

42 pairs of PCa specimens and matched normal prostate tissues from March 2018 to October 2019 were collected from the Department of Urinary surgery of the Affiliated Hospital of Yangzhou University (Yangzhou, China). PCa were confidently diagnosed by one pathologist. All fresh tissues were obtained during surgery and immediately stored in liquid nitrogen prior to use. Approval for this study was granted by the Institute Research Medical Ethics Committee of Affiliated Hospital of Yangzhou University (Yangzhou, China). Patients provided written informed consent.

### Cell lines culture

Human prostate cancer cell lines, PC-3 cells and LNCaP cells, were purchased from the American Type Culture Collection (Rockville, MD, USA). The cells were maintained in RPMI-1640 medium (Invitrogen Life Technologies), supplemented with 10% fetal bovine serum (FCS). The RWPE-1 (Human Prostate Epithelial Cell) cells were kindly provided by Dr X.Y. Dong (University of Yangzhou, China) and maintained in defined keratinocyte serum free medium with 5 ng / ml epidermal growth factor.

### Total RNA extraction and human Arraystar piRNA array

Total RNA was isolated from PCa tissues and matched normal prostate tissues as previously described [29]. The extracted RNA was submitted to Shanghai Kangcheng Bioengineering Technology Co., Ltd (Shanghai, China) for the Arraystar piRNA microarray screen and expression profiling analysis. The Human Arraystar piRNA array, which is designed for profiling 23000 human piRNAs (ArrayStar, Rockville, MD), was used for this study. Data analysis and image acquisition were provided by Shaghai Kangcheng Bioengineering Technology Co., Ltd (Shanghai, China).

### RNA in situ hybridization (ISH)

Tissue sections (thick 7 um) were dewaxed regularly and digested by pepsin to expose RNA. After RNAs were hybrided in 42°C for the night, the sections were added sealing fluid, biotinylated anti-digoxin, and streptavidin-biotin complex (SABC). Then biotin peroxidase was instilled. 3,3’-Diaminobenzidine tetrahydrochloride (DAB) was used for tissue staining. The sections were dyed hematoxylin, dehydrated and sealed. Morphological changes of tissue were observed under a microscope. Criteria for staining results: the brown and yellow particles in cytoplasm or nucleus were the positive result of piR-19166. Ten high-power fields were randomly selected to count the percentage of positive cells: less than 5% was low expression; more than 5% was high expression. A positive control group was set according to the reagent instruction, and the stained tissue without primary antibody was a negative control group.

### qRT-PCR analysis

Total RNAs from PCa cells, PCa tissues and matched normal tissues were isolated using TRIzol reagent (Invitrogen). RNAs were reverse-transcribed to cDNA using the PrimeScript First Strand cDNA synthesis kit (Takara) according to the instructions. qRT-PCR was performed on an Applied Biosystems 7500 Real Time PCR system. The expression of piRNA and mRNAs was normalized to U6 and GAPDH, respectively.

### PCa cells transient transfection and lentiviral infection

The assays were performed as previously described [13].

### Dual-luciferase reporter assay

The 167 nucleotide (nt) full length 3′-UTR of CTTN was amplified by PCR from genomic DNA of PC3 cells and cloned into the EcoRI and XhaI sites of pGL3-BS vector (Promega, WI, USA). The primers for CTTN 3′-UTR were as follows: 5′- tcggccaagaatactt -3′ and 5′-tcggtttgaatcatct -3′. The mutant 3′UTR of CTTN was generated using a Quick Change mutagenesis kit (Stratagene, Heidelberg, Germany) according to the manufacturer’s protocol. Co-transfection of piR-19166 or negative control and reporter vectors was performed using Lipofectamine 2000 (Invitrogen, San Diego, USA). After 48 h, dual luciferase activity was measured using a Dual-Luciferase Reporter Assay System (Promega, WI, USA) according to the instructions. Firefly luciferase signal was used for normalization.

### Transwell migration assay

For the migration assays, 2× 10^5^ cells were added into the upper wells transwell chamber without matrigel. In both assays, PC3 cells were seeded in culture medium without fetal bovine serum (FBS) in the upper wells, and in medium containing 10% FBS served as chemoattractant in the lower wells. After 14 hour incubation, the PC3 and LNCaP cells of the filters were fixed with methanol for 15 min, stained with 0.1% crystal violet for 20 min and counted in an inverted microscope (Olympus, Japan)

### Western blot array

The assay of western blot was performed as previously described [12]. The primary antibodies were as follows: anti- CTTN (Cat # ab33333, Abcam), and anti- MT1-MMP (Cat #ab3644, Abcam); anti-MMP2 (Cat #97779, Abcam), anti- MMP9 (Cat # ab38898, Abcam), and anti-GAPDH antibody (Cat #2118, CST). The secondary antibodies were horseradish peroxidase-conjugated anti-rabbit immunoglobulin-G antibody (Cat # ab6721, Abcam).

### Mouse xenograft and lung metastasis models

PC3 cells (5 × 10^6^) suspended by phosphate puffer solution (PBS) were injected subcutaneously into the backs of null mice (BABL/c, nu/nu, 23–27 g, 5–7 weeks of age, 10 mice/group) from Animal Center of Yangzhou University. The mice were randomly divided into the piR-19166 mimic group and NC mimic group. The tumor volume (length × width^2^/2) was used to measure with a slide caliper. For metastasis models, the PC3 cells were inoculated into the tail veins of 20 mice respectively. At the end of the arrays, the mice were scarified and the tumors and their lungs were removed, quantified and frozen for further assay. All nude mouse studies were approved in the animal facility at Ethics Committee on Animal Experimentation of Yangzhou University accordance with institutional guidelines.

### Immunohistochemistry (IHC)

The tumor specimens were fixed in formalin and dehydrated and embedded in paraffin wax. Then the specimens were deparaffinized using xylene liquid, rehydrated gradually, and repaired in antigen retrieval buffer. After move the buffer, the sections were incubated with relative primary antibody at 37°C for 2 h and then incubated with the second antibody at 25°C for 1 h. The DAB kit (MXB Biotechnologies, China) was used for staining immune complexes. The results of criterion showed that the tumor cell membrane and / or cytoplasm and / or nucleus appeared brown granules as positive. The percentage of positive cells was number of positive cells / all the cells in 5 random fields (100 cells in each field) under 10 × 40 high power microscope.

### Statistical analysis

SPSS V.16 software package for Windows (SPSS Inc., Chicago, IL, USA) was used for Statistical analyses and All graphs were created using GraphPad Prism 5 software (GraphPad Software, La Jolla, CA, USA). The student’s t-test was used to assess difference between groups. Chi-Square was applied for categorical variables. Correlation analysis was performed using the Spearman rank test. Statistical significant difference was considered as P<0.05.
